# Probing the role of an invariant active site His in family GH1 β-glycosidases

**DOI:** 10.1080/14756366.2019.1608198

**Published:** 2019-05-10

**Authors:** Andrea Strazzulli, Giuseppe Perugino, Marialuisa Mazzone, Mosè Rossi, Stephen G. Withers, Marco Moracci

**Affiliations:** a Department of Biology, University of Naples "Federico II", Complesso Universitario di Monte S. Angelo, Napoli, Italy;; b Task Force on Microbiome Studies, University of Naples Federico II, Naples, Italy;; c Institute of Biosciences and BioResources – National Research Council of Italy, Naples, Italy;; d Department of Chemistry, University of British Columbia, Vancouver, Canada

**Keywords:** Inhibition, reaction mechanism, glycosidases, extremophiles

## Abstract

The reaction mechanism of glycoside hydrolases belonging to family 1 (GH1) of carbohydrate-active enzymes classification, hydrolysing β-O-glycosidic bonds, is well characterised. This family includes several thousands of enzymes with more than 20 different EC numbers depending on the sugar glycone recognised as substrate. Most GH1 β-glycosidases bind their substrates with similar specificity through invariant amino acid residues. Despite extensive studies, the clear identification of the roles played by each of these residues in the recognition of different glycones is not always possible. We demonstrated here that a histidine residue, completely conserved in the active site of the enzymes of this family, interacts with the C2-OH of the substrate in addition to the C3-OH as previously shown by 3 D-structure determination.

## 1. Introduction

Family GH1 is one of the largest families of the carbohydrate active enzymes classification data bank CAZy (http://www.cazy.org ^1^), including more than 26,000 β-glycosidases, of which more than 300 have been characterised with the resolution of more than 60 3 D-structures solved and over 20 different substrates specificities identified. Functions of GH1 enzymes include the breakdown of oligosaccharides produced from the degradation of (hemi)celluloses of the plant cell wall by cellulolytic, Archaea, Bacteria, and fungi^2^ ^,^ ^3^ and aryl-glycoconjugates for defence mechanisms in plant − herbivore and plant − insect interactions^4^. In humans, deficiencies in GH1 lactase phlorizin hydrolase, which hydrolyses lactose in dairy products, is responsible for lactose intolerance^5^ while the neutral cytosolic β-glucosidase is a xenobiotic-metabolizing enzyme that hydrolyses flavonoid glucosides^5^. In addition, mutants of this family of enzymes, named glycosynthases, thioglycoligases, and thioglycosynthases, have been used for pioneering a novel approach for the chemo-enzymatic synthesis of oligosaccharides and still are the most common^6–16^.

GH1 enzymes follow the classical *retaining* reaction mechanism proposed by Koshland^17^. The catalytic acid/base and nucleophile of the reaction are a carboxylic acid and a carboxylate, respectively, with the exception of myrosinases where the former is replaced by a non-basic residue and hydrolysis occurs by substrate-assisted catalysis^18^. The substrate binding site includes, according to the current classification^19^, a -1 subsite, hosting the first sugar residue from the non-reducing end of the substrate, and ≥ +1 subsites (toward the reducing terminus of the substrate) binding the aglycone moieties. The residues involved in + n subsites are involved in substrate recognition, but not in catalysis. Instead, the amino acids forming the −1 subsite define the primary substrate specificity of the enzyme. GH1 includes enzymes hydrolysing gluco/galacto- and phosphogluco/galacto-, -manno, -xylo, -D-fuco, and –glucuronides.

The catalytic mechanisms and substrate binding sites of GH1 β-glycosidases have been the subject of extensive studies through enzymological characterisation performed with mechanism-based inhibitors and site-directed mutants, the inspection of 3 D-structures in the presence of substrate analogues and inhibitors, computational analysis, and quantum mechanics^20–25^. These approaches have helped define the roles of the invariant residues in the active sites of GH1 as either catalytic residues or in substrate binding. However, the role of an invariant His in the active site of these enzymes was not unequivocally assigned. According to computational studies on GH1 from rice, the highly conserved H130 does not always form a hydrogen bond with C3-OH^26^ Instead, cases in which the imidazole ring is inappropriately oriented to bind to this group or that can interact with both C2-OH and C3-OH have been reported^27^ ^,^ ^28^.

In this paper, we evaluated the function of this amino acid by combining site-directed mutagenesis, the use of mechanism-based inhibitors and thermodynamic cycles on the well studied β-glycosidase from *Sulfolobus solfataricus* (*Ss*β-gly). For this enzyme, which shows broad substrate specificity toward gluco-, galacto, and fucosides, high resolution 3 D-structures with different inhibitors and detailed enzymatic studies are already available^2^ ^,^ ^29–32^. Here we show that the removal of the imidazole ring of the invariant H150, though reducing the affinity of all substrates, did not affect the *k_cat_* values of aryl-glycosides, and, surprisingly, prevented the inhibitory effect of a mechanism-based inhibitor. The enzymatic characterisation of the A150 mutant allowed us to conclude that, at the transition state, the histidine helps mediate the ionic state of the nucleophile of the reaction (E387) and interacts with C2-OH, which could not be easily determined via the inspection of the 3 D-structure.

## 2. Experimentals

### 2.1. Reagents

All commercially available substrates were purchased from Sigma. Restriction endonucleases and T4 DNA ligase were from New England BioLabs (Ipswich, MA, USA). Synthetic oligonucleotides were from PRIMM (Milan, Italy).

### 2.2. Site-directed mutagenesis

The pGEX-2TK derivative (GE Healthcare) plasmid (pGEX-K-Gly) containing the wild type *S. solfataricus* β-glycosidase gene (*lacS*) was described previously^33^. The preparation of the *Ss*β-gly A150 mutant by site-directed mutagenesis from pGEX-K-Gly was performed by using the U.SE Mutagenesis Kit™ (GE Healthcare) with the following oligonucleotide (mutated codon is underlined): 5′-GAGGTAATGGCCATGCAT ACATGTTTAGTATAAAG-3′. The final plasmid containing only the desired mutation was confirmed by direct sequencing.

### 2.3. Enzyme expression and purification

Wild-type *Ss*β-gly and A150 mutant were expressed and purified as fusions of glutathione S-transferase (GST), as previously reported^33^. Briefly, transformed *E. coli* BL21(DE3)RIL cells, were grown in SB medium at 37 °C and induced by the addition of 1.0 mM IPTG when cultures reached an OD_600_ of 1.0. After 16.0 of incubation, cells were harvested by centrifugation at 5000 × *g* and stored at −20.0 °C. Then the pellet was thawed and resuspended in 2.0 ml/g cells of PBS 1X buffer (50.0 mM sodium phosphate buffer, 150.0 mM NaCl; pH 7.4). After French Press Cell disruption and centrifugation at 4.0 °C for 0.5 h at 30000 × *g* to remove cell debris, free cell crude extract containing the fusion protein was subjected to affinity chromatography on a glutathione-Sepharose 4B™ resin (GE Healthcare), followed by extensive washing with PBS 1X. Then, the 50% slurry resin was incubated at 4.0 °C for 16 h with thrombin protease, in order to cleave GST from *Ss*β-gly. The eluted fraction containing only the protein of interest was finally separated from the GST protein, which remained bound to the resin.

Aliquots of pure proteins (>95.0%, as judged by SDS-PAGE analysis) were stored at −20 °C in PSB 1X containing 20.0% glycerol. Samples stored in these conditions are stable for several months. The protein concentration was determined using the method of Bradford^34^ by using bovine serum albumin as standard.

### 2.4. Enzyme characterisation

All kinetic studies were performed by following spectrophotometrically the hydrolytic activity with a Cary 100 Scan spectrophotometer (Varian, Australia), coupled with a thermally controlled Peltier system. Reaction solutions (1.0 ml) were preheated for 2.0 min, keeping the temperature constant during all the measurements. The β-glycosidase activity performed at 65 °C in 50 mM sodium phosphate buffer at pH 6.5 was conventionally defined as the *standard reaction*. Kinetic parameters at standard conditions were determined by using artificial and natural substrates, whose concentrations were from 0.1 to 30.0 mM and from 1.25 to 750.0 mM, respectively. In order to correct for the spontaneous hydrolysis of the substrates, mixtures containing all the reactants except enzymes were prepared and referred as *blank* reactions. In each assay, amounts ranging from 0.5 to 20.0 μg of wild type *Ss*β-gly and A150 mutant were used. Measuring the hydrolytic activity on 2-nitrophenyl glycoside substrates under standard conditions, a molar extinction coefficient (ε_M_) value of 1711 M^−1 ^cm^−1^ at 405 nm was used, whereas the activity on lactose and cellobiose was determined by measuring the released glucose with the glucose oxidase-peroxidase enzymatic assay (GOD-POD), taking into account that one molecule of cellobiose leads to the release of two glucose units. One unit of enzyme activity was conventionally defined as the amount of enzyme which hydrolyses 1.0 μmol of the substrate in 1.0 min under standard conditions. All data were plotted and refined using the programme GraFit 5.0, in order to determine the steady-state kinetic parameters^35^.

Kinetic studies on 4-nitrophenyl β-D-glucopyranoside (4 Np-Glc) and 4-nitrophenyl 2-deoxy-β-D-glucopyranoside (4 Np-2d-Glc) were performed in standard conditions, by using a 0.1–30 mM substrate concentration range. In this case, 0.1 ml of reaction mixtures containing 0.5 μg of wild type *Ss*β-gly and A150 mutant were incubated in 1.5 ml tubes for 2.0 min, stopping the reaction with 0.9 ml of Na_2_CO_3_ 1.0 M. Activities were measured at 420 nm, using an ε_M_ value of 18,300 M^−1 ^cm^−1^, for the release of 4-nitrophenol.

The pH-dependence of hydrolytic activity of wild type *Ss*β-gly and A150 mutant was analysed using 4 Np-Glc at 65 °C in 50.0 mM sodium citrate (pH 3.0–5.6), sodium phosphate (pH 6.0–8.0) and KCl/borate (pH 8.0–10.0) buffers. Reactions were stopped with 0.9 ml of Na_2_CO_3_ 1.0 M, and activities were measured as above.

### 2.5. Thermal stability and temperature dependence

The thermal stability was determined by incubating all the enzymes at the concentration of 0.01 μ/mL in 50.0 mM sodium phosphate buffer pH 6.5, at the temperatures and times indicated. The hydrolytic activity of each sample was determined at 405 nm on 4 Np-Glc in standard conditions, using an ε_M_ value of 9 340 M^−1 ^cm ^−1^ for the release of 4-nitrophenol. The activity of the sample measured without any incubation was taken as 100%.

Enzymes were assayed in standard conditions in the 30–85 °C range of temperatures, in order to evaluate their temperature dependence. Enzymatic units were measured by using different ε_M_ values of 4-nitrophenol, as follows: 6130 M^−1 ^cm^−1^ (30 °C); 6570 M^−1 ^cm^−1^ (35 °C); 6910 M^−1 ^cm^−1^ (40 °C); 7530 M^−1 ^cm^−1^ (45 °C); 7970 M^−1 ^cm^−1^ (50 °C); 8420 M^−1 ^cm^−1^ (55 °C); 8890 M^−1 ^cm^−1^ (60 °C); 9340 M^−1 ^cm^−1^ (65 °C); 9700 M^−1 ^cm^−1^ (70 °C); 10,120 M^−1 ^cm^−1^ (75 °C); 10,610 M^−1 ^cm^−1^ (80 °C); 10,900 M^−1 ^cm^−1^ (85 °C).

### 2.6. Inhibition studies

This analysis was performed using the protocol described previously^7^. Briefly, 0.20 ml of 0.25 mg/mL (ca 1.0 μM) of the wild type *Ss*β-gly and the A150 mutant were incubated in 50 mM sodium phosphate buffer pH 6.5 at 50 °C for 16 h, in the presence of the Mechanism-Based Inhibitor (MBI) 2,4-dinitrophenyl 2-deoxy-2-fluoro-β-D-glucoside (2,4-DNp-2F-Glc) at 1000:1 inhibitor:enzyme molar ratio. 4.0 μL aliquots (ca 1.0 μg of enzyme) were taken before and after incubation to assay their hydrolytic activity at *standard* conditions, in the presence of 20.0 mM of 4 Np-Glc, and using an ε_M_ value of 9340 M^−1 ^cm^−1^ for the release of 4-nitrophenol. As a control, the same incubations and assays were performed in the absence of the inhibitor.

## 3. Results and discussion

### 3.1. Identification of substrate-binding determinants

**Figure 1.**
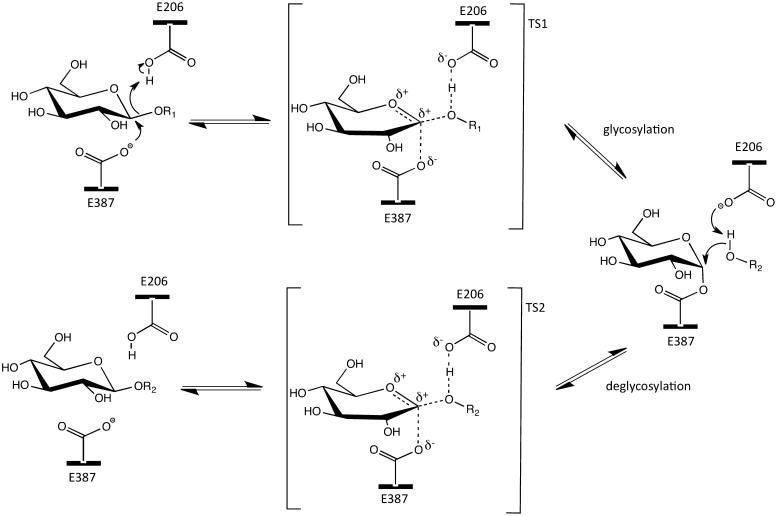
The retaining reaction mechanism of the β-glycoside hydrolase from *Sulfolobus solfataricus*. The highly conserved nucleophile and acid/base catalyst in the active site are indicated as E387 and E206, respectively. TS: transition state.

As is the case for the other GH1 β-glycosidases, *Ss*β-gly follows the classical *retaining* reaction mechanism proposed by Koshland. The hydrolysis of the glycosidic bond proceeds through a double displacement via two oxocarbenium-ion-like transition states involving a covalent glycosyl-enzyme intermediate (Figure 1). Catalysis is promoted by a couple of carboxylic acids in the active site working as acid/base nucleophile of the reaction, E206 and E387, respectively. In the first step of the reaction, or glycosylation step, glutamic acid 206 works as an acid donating a proton to the glycosidic oxygen while the nucleophile attacks the anomeric carbon leading to the departure of the aglycone group of the donor and to the formation of a covalent bond between the glycosyl group and E387 of the enzyme. In the second step, the same E206 residue works as a base activating the incoming water molecule that hydrolyses the glycosyl-enzyme intermediate leading to a product with the same anomeric configuration as the substrate.

**Figure 2.**
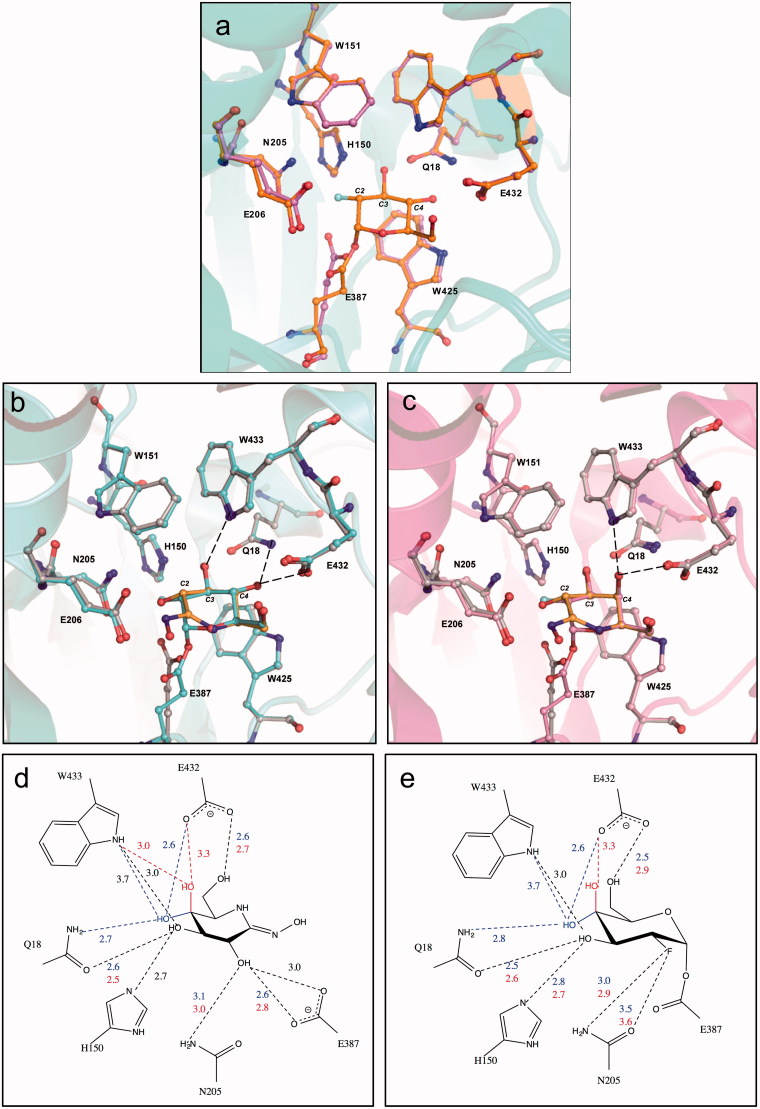
Inspection of the 3 D structure of *Ss*β-gly and the amino acid residues involved in the substrate binding. Seven residues (see Text) and the catalytic E206 and E387 are highlighted in *ball and stick* format. The 3 D structure of the *Ss*β-gly free (magenta) and in complex with 2 F-Glc orange) (a). Superimposition of the *Ss*β-gly 3 D structures complexed with 2 F-Glc (light blue) and D-glucohydroximolactam (orange) (b) and the corresponding *galacto*-based inhibitors (c) where the 2 F-Gal and D-galactohydroximolactam are in pink and yellow, respectively. All atoms are coloured by the CPK convention. Scheme of the interactions formed by the ligands TSA (d) and MBI (e) with the aminoacids of the -1 site of Ssβ-gly. Distances invariant in both ligands, and those specific for galacto- and glucoside configurations are indicated in black, red and blue, respectively.

The functional roles of E206 and E387 were demonstrated by site-directed mutagenesis and detailed characterisation of the mutants^33^. The inspection of the 3 D structure of *Ss*β-gly complexed with the *gluco*- and *galacto*- configured non-covalent Transition State Analogues (TSAs; D-gluco- and D-galactohydroximolactam) and the trapped covalent 2-deoxy-fluoroglycosyl-enzyme intermediates with the MBI 2 F-Glc and 2 F-Gal bound at the -1 site^31^ allowed the identification of the residues involved in sugar binding (Figure 2). A change in position of the catalytic nucleophile E387, which moves to accommodate the covalent linkage, was observed by comparing the structures of the unbound form of *Ss*β-gly and that complexed with 2 F-Glc (Figure 2(a)).

Despite the different conformations, ^4^H_3_ and ^4^C_1_ for TSA and MBI ligands, respectively, these compounds form similar interactions with the side chains of eight amino acids in the *Ss*β-gly substrate binding site, namely Q18, H150, W151, N205, W425, E432, W433, and the catalytic nucleophile E387 (Figure 2(b,c)). All are conserved among more than 5,000 GH1 family members while W151 and E432 are replaced by F and S, in 43% and 61% of cases, respectively. Tryptophans 151, 425, 433 make hydrophobic interactions with the four ligands, the C2-OH group interacts with E387 and N205, C3-OH with Q18 and the Nε2 of H150, while C6-OH hydrogen-bonds to E432 (Figure 2(d,e))^31^. The only difference in the interactions reflects the *gluco* and *galacto* configurations of the hydroxyl group at C4 of both TSA and MBI ligands (Figure 2(d,e), respectively). The axial C4-OH of galacto-configured ligands interacts with Oε2 of E432 and the nitrogen of W433. Instead, both the *gluco*-based inhibitors show two extra hydrogen-bonds, from C4-OH to the nitrogen of Q18 and between C3-OH and the nitrogen of W433 (Figure 2(d))^31^.

Among these residues, the side chains of Q18, N205, and H150 form a complex hydrogen-bonding network with the C3-OH and C4-OH of TSA and MBI ligands. H150 Nε2 atom is H-bonded to C3-OH, while N205 and Q18 are H-bonded to the hydroxyls of C2 and C4, respectively, with the latter also at a hydrogen bond distance from C3-OH (Figure 2(d,e)). To analyse the interactions with different ligand groups at the -1 site the H150 residue was thus mutated to alanine as follows.

### 3.2. Effect of H150 on C4-OH

**Figure 3.**
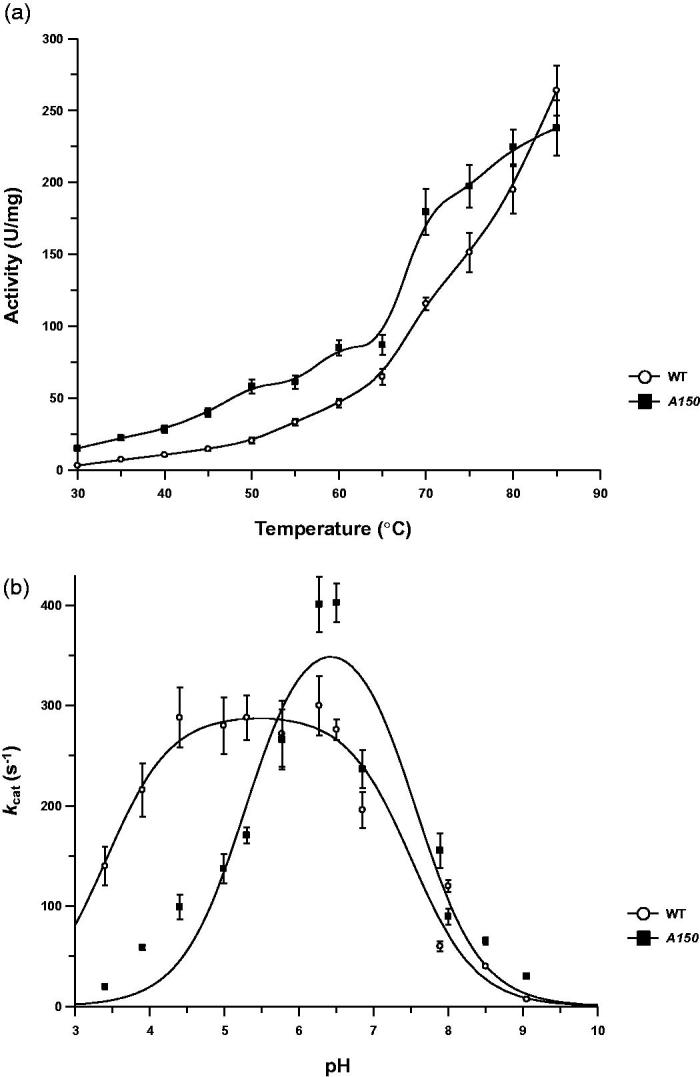
Effect of pH and temperature on wild type and mutant Ssβ-gly. Wild type *Ss*β-gly (closed circles) and the A150 mutant (open squares) specific activities are shown as a function of temperature (a) and pH (b). For the wild type and mutant, the pK_a_1 are 3.41 ± 0.13 and 5.29 ± 0.18, while the pK_a_2 are 7.50 ± 0.13 and 7.54 ± 0.20, respectively.

The specific activity of the A150 mutant on 4-nitrophenyl β-D-glucopyranoside (4 Np-Glc) increased continuously up to 85 °C exactly like the wild type (Figure 3(a)).

However, the *k_cat_* values of the two enzymes showed a different pH dependence, for the enzyme-substrate complex (Figure 3(b)). The A150 mutant showed sharper curve with an optimal pH of about 6.5 versus the broad pH_opt_ of the wild type (4.5–6.5) and changes in the acidic and basic limbs pK_a_ values of pK_a_ ^1^ ^^=3.41 to 5.29 and pK_a_ ^2^ ^^=7.51 to 7.54, respectively. The almost 2.0 units higher pK_a_ ^1^ in the A150 mutant indicates that at optimal pH 6.5 the nucleophile E387 in the enzyme-substrate complex exists more in the protonated state compared to the wild type, suggesting that the removal of the H150 imidazole ring could have altered the interactions between E387 and N205.

The steady-state kinetic constants of wild type and A150 on different substrates are summarised in Table 1. The general low catalytic efficiency (*k*_cat_/K_M_) of the mutant compared to the wild type is mainly due to its increased K_M_ values on all the substrates (from 3.2- to 27-fold), suggesting that the mutation loosened the interactions with the neighbouring residues in the catalytic site, reducing the affinity of the enzyme for the substrate.

**Table 1.** Steady-state kinetic constants at 65 °C of *Ss*β-gly wild type and A150 mutant.

	Wild type^a^			A150		
	K_M_ (mM)	*k*_cat_ (sec^−1^)	*k*_cat_/K_M_ (sec^−1^ mM^-1^)	K_M_ (mM)	*k*_cat_ (sec^−1^)	*k*_cat_/K_M_ (sec^−1^ mM^−1^)
2Np-Gal	0.9 ± 0.1	295.0 ± 6.3	310.0	24.7 ± 3.9	135.0 ± 15.1	5.5
2Np-Glc^b^	1.0 ± 0.2	538.0 ± 11.0	533.0	13.5 ± 2.2	1285.4 ± 124.7	95.2
Lactose	137.7 ± 6.2	710.8 ± 9.6	5.2	442.3 ± 62.7	35.8 ± 3.2	0.08
Cellobiose	33.20	274.7	8.3	549.9 ± 62.5	30.4 ± 1.9	0.06
4Np-Glc	0.94 ± 0.22	437.2 ± 23.4	439.4 K_1_^c^	23.94 ± 2.89	805.9 ± 53.0	33.7 K_2_
4Np-2d-Glc	6.87 ± 3.54	10.6 ± 1.9	1.5 K_3_	4.38 ± 0.88	44.2 ± 2.8	10.1 K_4_

^a^Data from^33^.

^b^For wild-type *Ss*β-gly from^7^.

^c^Specificity constant values used for the double-mutant cycle analysis method shown in Figure 4(b).

As previously reported, the catalytic efficiency of Ssβ-gly on disaccharides is lower than that on aryl glycosides, with the (*k*_cat_/K_M_)_2 Np-Gal_/(*k*_cat_/K_M_)_lactose_ and the (*k*_cat_/K_M_)_2 Np-Glc_/(*k*_cat_/K_M_)_cellobiose_ ratios of 60 and 137, respectively^2^, consistent with the better leaving group of the aryl glycosides. 2-Nitrophenol has a pK_a_ of 7.22 and is assisted by the *ortho effect* produced by the presence in position 2 of the phenol ring of the NO_2_ group^9^ ^,^ ^36^. Instead, in disaccharides, the high pK_a_ of 12.28 of glucose requires greater assistance by the acid/base in the glycosylation step of the reaction (Figure 1). Mutation affects more the (*k*_cat_/K_M_)_2 Np-Glc_/(*k*_cat_/K_M_)_cellobiose_ ratio (1.5 × 10^3^) while the ratio of the catalytic efficiencies on 2 Np-Gal and lactose is similar to the wild type (68 vs 60, for A150 and H150, respectively). The ratio of the catalytic efficiency of the mutant on 2 Np-Glc and -Gal is about 10-fold higher than that of the wild type with the (*k*_cat_/K_M_)_2 Np-Glc_/(*k*_cat_/K_M_)_2 Np-Gal_ being 17.3 and 1.7 for A150 and H150, respectively.

These data indicate that A150 mutant is more active on substrates showing an equatorial C4-OH (glucosides) rather than an axial hydroxyl at the -1 site, suggesting that the side chain of H150 is less important for galacto configured substrates. The distance of the imidazole ring of H150 from C4-OH of the sugars in both configurations is too far (5.5 Å) to be hydrogen-bonded (Figure 2(b,c)); thus, the mutation A150 might have produced a structural change in the enzyme or in the enzyme-substrate complex, which increased the turnover number on substrates with an equatorially configured of the C4-OH group. Alternatively, these interactions are mediated by, for example, Q18 (Figure 2). This is reminiscent of behaviour seen with the GH1 *Agrobacterium sp.* β-glucosidase Abg, wherein interactions with the equatorial 4-hydroxyl were shown to slow the deglycosylation step, such that the 4-deoxyglycoside actually hydrolysed faster than the parent substrate^37^.

### 3.3. Effect of H150 on C2-OH

To evaluate if the A150 mutation affects also the interaction with the hydroxyl group bound to C2, we tested on wild type and mutant the inhibitory effect of the 2,4-DNp-2F-Glc, the ligand used to obtain the 3 D-structure of *Ss*β-gly crystals shown in Figure 2 ^31^. The activity of the enzymes was measured at 65 °C in standard conditions on 20.0 mM 4 Np-Glc before and after incubation at 50 °C with 1.0 mM inhibitor (10^3^:1 inhibitor:enzyme molar ratio corresponding to 200:0.2 n moles). As expected, wild type almost completely lost its activity passing from 102 ± 8 U/mg to 5.88 ± 0.03 U/mg. Instead, the A150 mutant maintained >77% specific activity (from 85.2 ± 4.8 U/mg to 65.9 ± 2.7 U/mg) after 16 h incubation. In addition, surprisingly, under these conditions, A150 promoted the release of 92 n moles of 2,4-dinitrophenol from 2,4-DNp-2F-Glc while the wild type catalysed the hydrolysis of 4 n moles of product. Given that only 0.2 n moles of the enzymes were used, both enzymes must have been catalysing full turnover according to the following equation:

(1) $$E+2,4-\text{DNp}-2F-\text{Glc}\overset{k_{1}}{\underset{k_{-1}}{⇌}}E:2,4-\text{DNp}-2F-\text{Glc}\overset{k_{2}}{\to }E-\text{Glc}-2F\overset{k_{3}}{\to }E+\text{Glc}-2F$$

Interestingly A150 catalysed this turnover 23-fold faster than did the wild type, indicating that the mutant recognised 2,4-DNp-2F-Glc as a poor substrate, rather than a true inhibitor. Examples of GHs that remain catalytically competent or that are partially inhibited by MBI have been reported previously^38^ ^,^ ^39^. According to Equation (1), this indicates that the *k_3_* of the reaction catalysed by A150, although low, is much higher than that of the wild type. Presumably, alanine destabilised the covalent E-Glc-2F intermediate to an energy status higher than that occurring in the wild type catalysed reaction. An alternative explanation is that the other interpretation is that the TS for this step is lower for the Ala mutant, again consistent with the interactions at the equatorial 4-position^37^. This might facilitate the formation of the second transition state and the completion of the deglycosylation step (Figure 1). These results indicate that the A150 mutation induced a structural change in the active site of *Ss*β-gly involving groups interacting with C2-OH. The 3 D-structures clearly showed that H150 is slightly too far from the C2OH in TSA and the C2F in MBI ligands (3.5 and 3.3 Å for D-galacto- and D-glucohydroximolactam and 3.2 Å for both 2-deoxy-fluorogalacto- and glucoside, respectively). This prompted us to further investigate the nature of the interaction between the H150 and the C2-OH group of the substrate by using the method of double-mutant cycles (for a review see^40^).

### 3.4. Double-mutant cycles of H150 and the C2-OH

**Figure 4.**
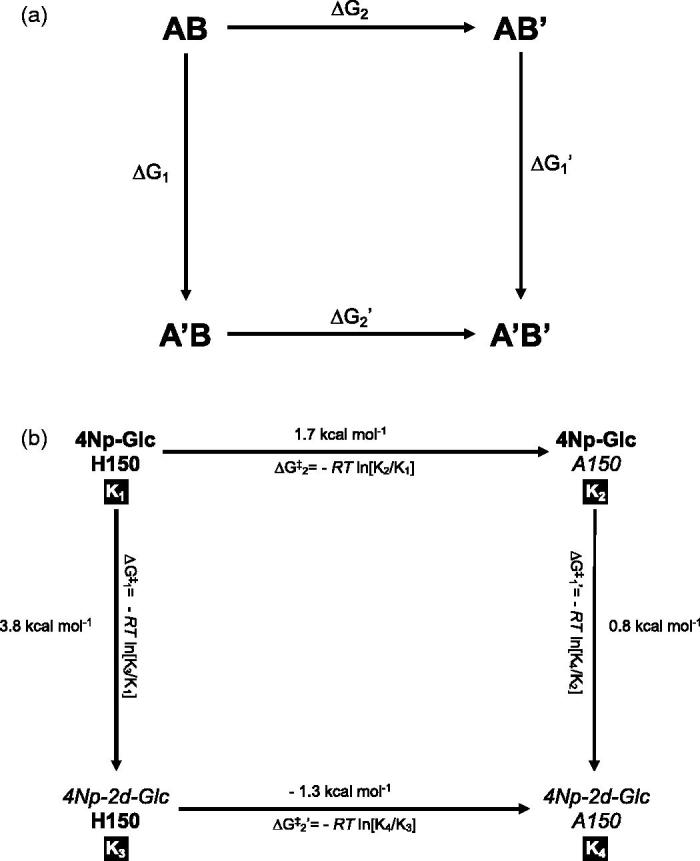
Double-mutant cycle analysis. *k*_cat_/K_M_ values correspond to K_1_−K_4_ values in Table 1 as follows: K_1 _= (*k*_cat_/K_M_)_H150→4NpGlc_; K_2 _= (*k*_cat_/K_M_)_A150→4NpGlc_; K_3 _= (*k*_cat_/K_M_)_H150→4 Np-2d-Glc_; K_4 _= (*k*_cat_/K_M_)_A150→4 Np-2d-Glc_. Mutations are highlighted in *italics*. By applying Equation (3), ΔG^‡^ values were calculated, leading to the final determination of the coupling energy |ΔG^‡^_1_−ΔG^‡^_1_′| (=|ΔG^‡^_2_−ΔG^‡^_2_′|), as indicated in the text.

Double mutants cycles were developed for analysing functional enzyme-ligand and protein folding interactions^41–43^. Figure 4(a) shows that a double-mutant cycle compares the difference in variation of free energy (ΔG) between wild type (AB), two single mutants (A′B and AB′, respectively), and their double mutant (A′B′). ΔG_1_ is the ΔG change for the A→A′ mutation, while the ΔG_2_ value derived from the B→B′ mutation. If two groups do not interact with each other, the effect of both mutations will be independent and additive:

$$\Delta G_{1}=\Delta {G^{\prime }}_{1}\text{and}\Delta G_{2}=\Delta {G^{\prime }}_{2}$$

Instead, if the effects of the mutations are not independent on each other, the effect of the modification of one group may be different, depending on the presence or not of the other group, then:

$$\Delta G_{1}\neq \Delta {G^{\prime }}_{1}\text{and}\Delta G_{2}\neq \Delta {G^{\prime }}_{2}$$

The free energy of coupling (ΔΔG) between residues A and B is given by:

$$\left|\Delta G_{1}-\Delta {G^{\prime }}_{1}\right|=\left|\Delta G_{2}^{{}^{‡}}-\Delta G_{2}^{{}^{‡}}{}^{\prime }\right|\neq 0$$

We applied the double-mutant cycles approach to compare the ΔG values obtained with 4 Np-Glc and its 2-deoxy analogue 4 Np-2d-Glc, which differs in the presence and absence of hydroxyl group at the C2 (A→A′ mutation) and wild type and the A150 mutant (B→B′ mutation). We, therefore, considered H150 and 4NpGlc as ‘wild types’, A150 and 4 Np-2d-Glc as ‘single mutants’ and the combination of those as ‘double mutants’.

The free energy values were calculated from the *k*_cat_/K_M_ obtained experimentally, by the use of the equation:

(2) $$\Delta G^{‡}=-\mathrm{RT}\;\text{ln}\;\;(k_{\text{cat}}{/K}_{M})$$

where *R* is the gas constant (1.987 cal K^−1 ^mol^−1^) and *T* is the absolute temperature (298 K).

The *k*_cat_/K_M_ values for the mutant with the wild type enzymes measured on 4NpGlc and 4 Np-2d-Glc (Table 1) allow calculation of the free energy values in Figure 4(b) by using the equation:

(3) $$\Delta G^{‡}\;=\;\mathrm{RT}\;\text{ln}\;\;[{\left(k_{\text{cat}}{/K}_{M}\right)}_{\text{mut}}/{\left(k_{\text{cat}}{/K}_{M}\right)}_{\text{wt}}]$$

In this way, we deduced quantitatively how the mutation affects the interaction energy of the enzyme with the transition state^41^. As can be seen in Figure 4(b):

$$\Delta G^{‡}{}_{1}\;\neq \;\Delta G^{‡}{{}_{1}}^{\prime }\text{and}\Delta G^{‡}{}_{2}\neq \;\Delta G^{‡}{{}_{2}}^{\prime }$$

Indicating that mutations are not independent of each other. In other words, the removal of the imidazole ring of H150 is different if the hydroxyl group at the C2 is present or not in the substrate.

The calculated coupling energies were:

$$|\Delta G^{‡}{}_{1}-\;\Delta G^{‡}{{}_{1}}^{\prime }|\;=\;3.00\text{kcal}/\text{mol}$$

$$|\Delta G^{‡}{}_{2}-\;\Delta G^{‡}{{}_{2}}^{\prime }|\;=\;3.00\text{kcal}/\text{mol}$$

These values, being different from zero, indicating that an interaction between the imidazole of H150 of *Ss*β-Gly and the C2-OH group on pyranose ring of the substrate occurs when wild type enzyme catalyses the hydrolysis of 4 Np-Glc. It is worth mentioning that the values of net binding energy are similar to those measured with the same approach on enzymes from mesophiles indicating that the stability of the protein is not relevant.

Available 3 D structural data clarified that H150 interacts with C3-OH, while kinetic data strongly indicated that the imidazole group of this residue is also involved in the stabilisation of the hydroxyl groups of the C4 and C2 of the pyranose ring. In particular, the lack of inhibition with a MBI carrying a fluorine atom at C2, and the double-mutant cycles, strongly support interaction with the C2-OH of the substrate. Indeed, the inspection of the 3 D-structure of Ssβ-gly clearly shows that H150 lies between residues N205 and Q18 in the close vicinity of C2- and C3-OH (2.8 and 3.3 Å, respectively). Instead, H150 is farther from C4-OH: it is possible, therefore, that the different specificity of A150 on gluco- and galactosides are the result of structural rearrangements in the active site rather than direct contact with the C4-OH group. The functional characterisation suggests that H150 not only interacts with C3-OH group as indicated by crystallographic data, but it might also interact with the C2-OH group.

## 4. Conclusions

We reported here that combining the inspection of high-resolution 3 D-structure complexes, the rational modification of an amino acid residue in the active site, and the functional comparison of wild type and mutant, allows us to reveal previously unknown enzyme-substrate interactions in a GH1 glycoside hydrolase.
